# Prevalence of metabolic syndrome in sickle cell disease patients: A cross‐sectional study at a tertiary hospital in Nepal

**DOI:** 10.1002/jha2.989

**Published:** 2024-08-05

**Authors:** Ramesh Khadayat, Mukesh Bishwakarma, Shubham Pant, Om Prakash Bhatta, Pariwesh Raj Bista, Sher Bahadur Kamar

**Affiliations:** ^1^ Patan Academic of Health Sciences Patan Hospital Kathmandu Nepal; ^2^ Seti Provincial Hospital Dhangadi Nepal; ^3^ Tribhuvan University Institute of Medicine Maharajgunj Medical Campus Maharajganj Bagmati Nepal; ^4^ WHO Nepal Kathmandu Nepal

**Keywords:** dyslipidemia, metabolic syndrome, Nepal, obesity, sickle cell disease

## Abstract

**Background:**

Sickle cell disease (SCD) is the most common hemoglobinopathy caused by an autosomal recessive genetic disorder leading to increased morbidity and mortality rates. SCD is prevalent in the Tharu community in the lowland (Terai) region of Nepal. Prevalence of metabolic syndrome among adults with SCD is poorly studied.

**Methodology:**

This prospective cross‐sectional study was conducted at Seti Provincial Hospital in Dhangadhi, Nepal, among 140 adolescents and adults with SCD, aged 15–60 years. Anthropometric and laboratory data were collected using an assisted questionnaire, and the SPSS software version 23 was used for data analysis. Descriptive and inferential statistics were used to summarize the presence of metabolic syndrome and were stratified in separate analyses by age and sex. National Cholesterol Education Program‐Adult Treatment Panel III criteria were used to define metabolic syndrome.

**Aim:**

This cross‐sectional study aimed to assess the prevalence of metabolic syndrome among SCD patients with SCD registered at the Seti Provincial Hospital in Dhangadhi, Nepal.

**Results:**

The prevalence of metabolic syndrome in the study participants was 7.8%. Our study revealed 5% of the patients overweight, and 1.4% obese. In this study, the mean triglyceride level was 118.5 mg/dL, and the mean high‐density lipoprotein (HDL) level was 36.2 mg/dL (men) and 36.7 mg/dL (women). This study found that the mean fasting blood glucose level was 88.6 gm/dL. Similarly, 3.5% of patients had increased systolic blood pressure, and 7.8% had raised diastolic blood pressure. Study shows that changes in triglyceride level (*p* = 0.013), waist circumference, and HDL level (*p* = 0.0001 and 0.0048, respectively) are significantly associated with smoking or alcohol consumption; however, change in blood pressure (*p* = 0.013) and fasting blood sugar level (*p* = 0.086) are not associated with smoking or alcohol consumption.

**Conclusion:**

Study concluded that though a lower proportion of SCD patients met the criteria for metabolic syndrome than in studies conducted in developed countries, it is crucial to consider metabolic syndrome while managing patients with SCD. Nevertheless, the authors advocate a more comprehensive study to draw significant conclusions.

**Abbreviations**
BMI: body mass index
BP: blood pressure
CDC: Centers for Disease Control and Prevention
CVD: cardiovascular disease
HDL: high‐density lipoprotein
LDL: low‐density lipoprotein
NCEP ATP III: National Cholesterol Education Program Adult Treatment Panel III
SCD: sickle cell disease
TC: total cholesterol
TG: triglyceride

## 1 INTRODUCTION

Sickle cell anemia (SCD) is a prevalent genetic condition. It is an autosomal, recessive hemoglobinopathy characterized by hemolytic anemia, intermittent occlusion of small vessels leading to acute and chronic tissue ischemia, and multiorgan dysfunction [1, 2]. It is caused by inherited mutant hemoglobin genes resulting from a specific point mutation in the beta chain of hemoglobin, causing a missense substitution of valine for glutamic acid at position 6. SCD is the most common hereditary hemoglobinopathy. Under low oxygen tension, acidosis, and dehydrating conditions, biconcave disc‐shaped red blood cells (RBCs) change to a sickle shape because of polymerization of the defective hemoglobin (HbS), leading to intravascular hemolysis and vaso‐occlusion [3, 4].

SCD is characterized by significant hemolytic anemia, vascular dysfunction, and pulmonary hypertension. Hemolysis in SCD and related disorders is associated with high reactive oxygen species due to higher autoxidation of HbS, generating superoxide anion radicals and hydrogen peroxide, significantly affecting serum blood glucose and lipid profile, and leading to vascular dysfunction. These will result in insulin resistance and atherogenesis and increase the rates of metabolic syndrome with its complications [3, 5].

This condition affects millions of people worldwide, particularly those of African, Mediterranean, Middle Eastern, and South Asian descent. Additionally, the indigenous Tharu ethnic group inhabiting the Terai (lowland) region of Nepal has been shown to have a high prevalence of sickle cell disease (SCD), with previous studies showing a prevalence of 9.3% for sickle cell trait in this group [6].

Metabolic syndrome is a cluster of disorders associated with an increased risk of diabetes mellitus and cardiovascular diseases (CVD). It is characterized by central obesity, hypertriglyceridemia, low high‐density lipoprotein (HDL) cholesterol levels, hyperglycemia, and hypertension. The NCEP ATP III (National Cholesterol Education Program Adult Treatment Panel III) criteria require the presence of three or more of the following features: central obesity, hypertriglyceridemia, low HDL cholesterol, hypertension, and elevated fasting plasma glucose [7, 8]. According to the Centers for Disease Control and Prevention (CDC), body mass index (BMI) is classified into four categories: less than 18.5 underweight, 18.5–24.9 normal, 25–30 overweight, and greater than 30 obese. Patients with metabolic syndrome are at an elevated risk of developing CVD and other obesity‐related disorders, leading to increased morbidity and mortality [9, 10]. However, little is known about the prevalence of metabolic syndrome determinants in adults with SCD.

In Nepal, national studies on metabolic syndrome in people with SCD are essential for allocating resources, developing focused treatments, and comprehending its impact on the SCD community. The results of this study will provide information for evidence‐based policies and recommendations.

## 2 METHODS

### 2.1 Study design and setting

A prospective cross‐sectional descriptive study design was used to evaluate 140 adolescents (more than 10 years old) and adult patients with SCD (aged between 10 and 65 years), who were selected during the data collection for 6 months starting March 1, 2023, at Seti Provincial Hospital, a tertiary‐level referral hospital with a patient base from nine neighboring districts. Situated in a region with a high prevalence of sickle cell anemia among the Tharu ethnicity in Nepal, it is an ideal center for this study. This study was approved by the Ethics Committee of the Nepal Health Research Council on February 2, 2022. Written informed consent was obtained from all patients at the time of recruitment and before the first data collection. The NCEP ATP III criteria for diagnosis of metabolic syndrome and CDC classification were used to classify BMI (Table 1).

**TABLE 1:** Demographics of study participants.

		Sex		
		Male	Female	Total
Age	10–17	14	17	31
	18–60	47	61	108
	60+	0	1	1
Occupation	Agriculture	24	37	61
	Business	7	2	9
	Housewife	0	11	11
	job	2	2	4
	Labor	9	2	11
	Student	19	25	44
Body mas index (BMI) range	<18.5	20	29	49
	18.5–24.9	37	45	82
	25–29.9	3	4	7
	>30	1	1	2

### 2.2 Data collection

Data were collected from patients visiting Seti Provincial Hospital by taking interviews with informed consent and observing the blood reports (Table 2).

**TABLE 2:** Criteria for defining metabolic syndrome according to NCEP ATP III.

Risk factors	Defining levels
**Waist circumference**	Men: >102 cm/40 inches Women: >89 cm/35 inches
**Blood pressure**	Systolic: ≥130 mmHg Diastolic: ≥85 mmHg
**HDL**	<40 mg/dL (1.03 mmol/L) in men <50 mg/dL (1.3 mmol/L) in female
**Triglycerides**	> 150 mg/dL
**Fasting blood glucose**	>110 mg/dL

Abbreviations: HDL, high‐density lipoprotein; NCEP ATP III, National Cholesterol Education Program Adult Treatment Panel III.

### 2.3 Inclusion and exclusion criteria

Only patients with SCD were included in the study. Patients with compound heterozygosity (sickle/beta thalassemia), concurrent alpha thalassemia, hereditary spherocytosis, thalassemia, and congenital heart disease were excluded from the study.

### 2.4 Statistical analysis

Descriptive analysis of the independent variables was performed in terms of frequency and percentage. Data were collected using data collection forms and then logged into Microsoft Excel. After data were entered in Excel, they were exported into SPSS format, and frequencies, cross‐tabulation, and percentages were analyzed using IBM SPSS 23 software. The chi‐square test was performed at the 5% significance level to calculate the association between the study variables.

## 3 RESULTS

**FIGURE 1:**
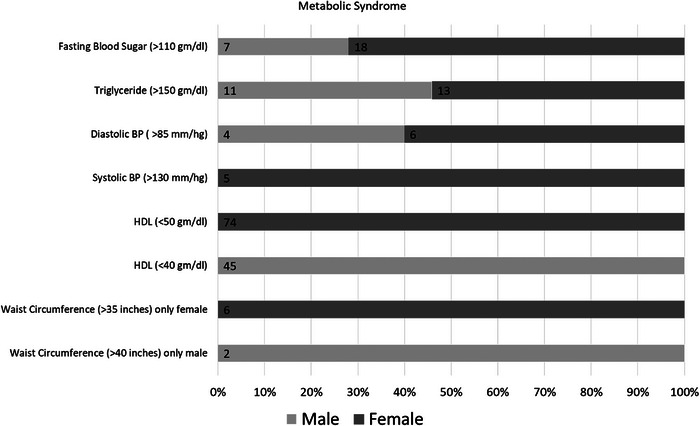
Metabolic syndrome criteria among males and females.

**FIGURE 2:**
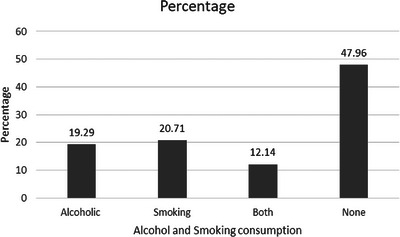
Alcohol and smoking intake among study participants.

This study was conducted with 140 participants, comprising 79 females and 61 males, all of whom were SCD patients from the Tharu communities. As shown in Figure 1, males and females aged 10–17 years were 14 and 17, respectively. However, in the age group of 18–60 years, there were 47 male SCD patients and 61 female patients. In the age group above 60 years, we had only one female patient and no male patients. A total of 140 SCD patients were included in the study, of which 44 were students. Of the 44 students in our study, eight females and 10 males between the ages of 1 and 15 attend primary and lower secondary schools, while nine males and 17 females between the ages of 15 and 25 are largely enrolled in secondary schools and higher education institutions. The most common occupation among the study participants was agriculture, followed by labor, housewife, business, and government or non‐government job holders, as seen in Figure 2.

Among our study patients, we found that 58.57% were classified as having a normal BMI, 35% were underweight, 5% were overweight, and 1.43% were obese (Table 3).

**TABLE 3:** Association between alcohol and/or smoking with metabolic syndromes criteria.

Value	Alcoholic and/or smoker	Non‐alcoholic or smoker	Chi‐square value (*Χ* ^2^)
**TG >150 mg/dL**	18	6	6.16, *p* = 0.013
**TG <150 mg/dL**	55	61	
**BP >130/85**	8	3	2.02, *p* = 0.154
**BP <130/85**	65	64	
**Waist circumference >35** **inches**	5	1	53.007, *p* = 0.0001
**Waist circumference <35 inches**	1	72	
**FBS >100 gm/dL**	17	8	2.93, *p* = 0.086
**FBS <100 gm/dL**	56	59	
**HDL <50 mg/dL**	68	51	7.94, *p* = 0.0048
**HDL >50 mg/dL**	5	16	

Abbreviations: BP, blood pressure; FBS, fasting blood sugar; HDL, high‐density lipoprotein; TG, triglyceride.

Figure 1 presents the metabolic syndrome criteria value among males and females. Among the total male participants from the study, four males (6.5%) have three or more criteria for symptoms of metabolic syndrome. And among the total female participants from the study, seven females (8.8%) have three or more criteria symptoms of metabolic syndrome, as shown in Table 4. Overall, among 140 participants, 11 patients (7.8%) have three or more criteria for metabolic syndrome.

**TABLE 4:** Patients fulfilling three or more criteria of metabolic syndrome.

Metabolic syndrome criteria	Male	Female
**≥3 symptoms**	4 (6.5%)	7 (8.8%)
**<3 symptoms**	57 (93.5)	72 (91.2%)

In our study, we found that among our study participants, 20.7% were smoking, and 19.2% were involved in alcohol intake. While 12.1% of study participants were involved in both smoking and alcohol, and 47.8% were not taking either alcohol or smoking as seen in Figure 2.

In this study, we calculated the association between alcohol and/or smoking intake and metabolic syndrome criteria using a non‐parametric test (chi‐square, *Χ* ^2^) at a 5% level of significance. We found that the change in triglyceride (TG) levels is statistically associated with the intake of alcohol or smoking (*p* = 0.013) at a 5% level of significance. However, our study concluded that the change in blood pressure (BP) is not significantly associated with alcohol or smoking intake (*p* = 0.154). Waist circumference and HDL were found to be statistically associated with the intake of alcohol or smoking (*p* = 0.0001 and 0.0048, respectively) with a 95% confidence interval. According to our collected data, fasting blood sugar was not associated with smoking or alcohol intake (*p* = 0.086).

## 4 DISCUSSION

SCD is a condition that affects multiple organ systems, affects quality of life, and contributes to increased morbidity and mortality in affected patients. The life expectancy of patients with SCD has increased owing to earlier diagnosis and improved care, especially in developed countries. Infection prevention and pain management have enhanced their quality of life [11]. However, this increased longevity has led to more long‐term complications, including metabolic and endocrine disorders, in the SCD population. Appropriate treatment of these complications is vital for prognosis and quality of life. Individuals with SCD are at risk of CVD and subsequently develop various related complications. Consequently, CVD has become the most common cause of overall mortality among SCD patients [12, 13].

The authors conducted a study to examine the prevalence of metabolic syndrome among individuals with SCD in a tertiary care center in the western part of Nepal, which is one of the most affected areas and one of the least developed. This study could raise awareness among policymakers about the gravity of the situation and inspire broader research on the topic, ultimately leading to better patient management and an improved quality of life.

A total of 7.8% of the patients (male 6.5% and female 8.8%) in the study met the criteria for metabolic syndrome, which is lower than the prevalence reported in other studies. The reasons behind the decreased prevalence could be multifactorial such as socioeconomic factors, occupation, and lifestyle [14]. As no similar studies have been reported in Nepal, and there is a paucity of published literature on this topic from developing and resource‐limited settings, comparisons with other studies could not be made. Metabolic syndrome was defined according to clinical and biochemical parameters using the National Cholesterol Education Program‐Adult Treatment Panel III criteria [8].

A similar study conducted in an urban academic medical center in the United States showed that 14.3% of adults with SCD met the criteria for metabolic syndrome, with no difference in prevalence between males and females [15].

In an Italian cohort of children and adolescents with SCD comprising 52 patients, 48/52 patients (92%) had at least one metabolic and/or endocrine alteration: insufficiency/deficiency of vitamin D (84.7%), insulin resistance (11.5%), growth hormone deficiency (3.8%), subclinical hypothyroidism (3.8%), and hypogonadism (1.9%) [16].

The mechanism of obesity in SCD is complex and is likely influenced by many variables. Obesity in SCD may result from treatment advances (hydroxyurea and transfusions), increasing obesogenic environments, and a shift to a more sedentary lifestyle [17, 18]. Our study revealed that 5% of the patients were overweight and 1.43% were obese, which was also lower than that reported in other similar studies. This might also be influenced by socioeconomic factors, as the most common occupations, such as agriculture and manual labor, could influence the prevalence of metabolic disorders due to physical work and poor socioeconomic conditions. Another noticeable aspect was the higher prevalence of undernutrition compared to obesity, which contradicts findings from similar studies conducted in developed countries, but is similar to studies from low‐ and middle‐income countries [19, 20]. This highlights the influence of socioeconomic status and occupation on the prevalence of metabolic syndrome in SCD patients.

The discrepancy in results might be due to differences in the study design (longitudinal vs. cross‐sectional). The mechanism of obesity in SCD is complex and is likely influenced by many variables. Obesity in SCD may result from treatment advances (hydroxyurea and transfusions), increasing obesogenic environments, and a shift to a more sedentary lifestyle [17, 21].

Our cohort showed a significant proportion of individuals involved in smoking and alcohol consumption, which might contribute to the already increased CVD risk factors, as shown in previous studies [18].

In our SCD population, a small percentage of males and females met the criteria for metabolic syndrome, in contrast to studies from developed nations. There is a paucity of published literature on this topic from developing and resource‐limited settings.

A significant proportion of our patients showed dyslipidemia, which might be related to their diet and lifestyle. One of the criteria for diagnosing metabolic syndrome is dyslipidemia: TG ≥150 mg/dL or HDL less than 40 mg/dL (male), less than 50 mg/dL (female). In this study, the mean TG level was 118.5 mg/dL, which is low to be a risk factor for metabolic syndrome. The mean HDL level was 36.2 mg/dL (men) and 36.7 mg/dL (women), which could be considered a risk factor for metabolic syndrome. Only 26.2% of males have HDL levels ≥40 mg/dL. This study found that the mean fasting blood glucose level was 88.6 gm/dL.

Similarly, 3.5% of patients had raised systolic and 7.8% had raised diastolic BP. Studies done in other settings have shown a higher prevalence of hypertension [15].

In a prospective observational study from India involving 50 cases and controls, patients with SCD exhibited significant metabolic alterations compared with controls. Total cholesterol (TC) levels were notably lower in SCD patients, with a mean TC of 211 mg/dL compared to controls (*p* = 0.001). HDL levels were substantially reduced in SCD patients (mean HDL = 33.74 mg/dL), while a few patients had elevated HDL levels (mean HDL = 105.6 mg/dL, *p* = 0.001). Additionally, TG levels were significantly higher in patients with SCD (*p* = 0.01). Moreover, patients with SCD demonstrated elevated low‐density lipoprotein (LDL) levels, with a statistically significant increase (*p* = 0.01) [22].

There was wide variability in prevalence, which could be attributed to the study settings. This variability is observed in developed and developing countries, and is likely due to differences in lifestyle and socioeconomic factors. Although learning about the prevalence of metabolic syndrome will help prioritize the issue for concerned stakeholders, it is pivotal to conduct similar studies on a larger patient sample to draw significant conclusions and direct necessary policy‐level interventions.

## 5 STRENGTHS AND LIMITATIONS

Being one of the only studies of its kind from Nepal, this research opens new avenues for further exploration of the topic, stimulates awareness, and encourages subsequent studies by fellow researchers in the field. A sample size of 140 patients, though not sufficient for drawing solid conclusions, is still adequate to synthesize some evidence regarding the necessity for larger and more comprehensive studies.

We aimed to obtain a more extensive dataset to generate conclusive evidence. However, they face challenges owing to financial, time, and resource constraints. Additional difficulties arose from the lack of a proper follow‐up system in the hospital, and because most patients had poor financial conditions and lower literacy levels, many were lost to follow‐up. Not all confounders could be addressed because of resource limitations, as was the case with detailed biochemical investigations focused on the metabolic profile.

## 6 CONCLUSIONS

In conclusion, this cross‐sectional study, conducted at a tertiary center in western Nepal, revealed a prevalence of 7.8% metabolic syndrome among patients with SCD. Despite its relatively lower prevalence compared with other studies, these findings emphasize the importance of considering metabolic syndrome in the context of SCD management. Further research is warranted to unravel the multifaceted relationship between SCD and metabolic syndrome, thereby enhancing our understanding of potential interventions and strategies for improved patient care.

## AUTHOR CONTRIBUTIONS

**Mukesh Bishwakarma** and **Sher Bahadur Kamar**: study concept; data collection; and management of the patient. **Ramesh Khadayat**; **Mukesh Bishwakarma**; **Om Prakash Bhatta** and **Shubham Pant**: writing—original draft preparation and editing. **Sher Bahadur Kamar**: senior author and manuscript reviewer. All authors critically reviewed; revised; and contributed to the article. All authors read and approved the final version of the manuscript.

## CONFLICT OF INTEREST STATEMENT

The authors declare no conflicts of interest.

## FUNDING INFORMATION

Authors did not receive any specific grant from funding agencies in the public, commercial, or not‐for‐profit sectors.

## ETHICS STATEMENT

This study was conducted following review and approval by the ERB of NHRC. Consent was obtained from all participants.

## PATIENT CONSENT STATEMENT

The authors have confirmed patient consent statement is not needed for this submission.

## CLINICAL TRIAL REGISTRATION

The authors have confirmed clinical trial registration is not needed for this submission.

## Data Availability

Data used and analyzed during the current study are available from the corresponding author upon reasonable request.
